# Foods, Dietary Patterns and Occupational Class and Leukocyte Telomere Length in the Male Population

**DOI:** 10.1177/1557988317743385

**Published:** 2017-12-06

**Authors:** Behrooz Karimi, Ramin Nabizadeh, Masud Yunesian, Parvin Mehdipour, Noushin Rastkari, Afsaneh Aghaie

**Affiliations:** 1 Department of Environmental Health Engineering, School of Public Health, Arak University of Medical Sciences, Arak, Iran; 2 Department of Environmental Health Engineering, School of Public Health, Tehran University of Medical Sciences, Tehran, Iran; 3 Department of Research Methodology and Data Analysis, Institute for Environmental Research (IER), Tehran University of Medical Sciences, Tehran, Iran; 4 Department of Medical Genetics, School of Medicine, Tehran University of Medical Sciences, Tehran, Iran; 5 Center for Air Pollution Research (CAPR), Institute for Environmental Research (IER), Tehran University of Medical Sciences, Tehran, Iran; 6 Blood Transfusion Research Center, High Institute for Research and Education in Transfusion Medicine, Tehran, Iran

**Keywords:** foods groups, dietary patterns, occupational class, serum lipids, telomere length

## Abstract

Telomeres contain TTAGGG repetitive sequences and are located at the end of human chromosomes. Telomere dysfunction is associated with some age-related and chronic diseases, but its relationship with foods, dietary patterns, and occupational class in the young male population is not yet known. In this cross-sectional study, 300 healthy men, residents of Tehran, aged 25–40 years were enrolled from January to December 2016. We employed a cross-sectional study of 300 healthy people, residents of Tehran, aged 25-40 years. A food frequency questionnaire was used to obtain food intakes of all participants and converted into actual food intake (g/day). The principal components analysis was used to determine dietary patterns and other demographic characteristics. Leukocyte telomere length (TL) was measured by quantitative real-time polymerase chain reaction (PCR) to measure number of telomere repeat copy number (T) to the relative number of 36B4 copies (S) (T/S ratio). T/S in office-workers, waste recyclers, and other workers were 1.22 ± 0.4, 1.08 ± 0.3, and 1.094 ± 0.34, respectively. The results of multivariate linear regression adjusted for age, body mass index (BMI), and smoking were showed that whole grains (β = 0.02; *p* = .05), refined grains, fruits and vegetables, fish and dairy products were associated with an increase in log-T/S, but consumption of nuts and seeds (β = −0.00072; *p* = .06), meats (β = −0.00043; *p* = .9), produced meats (β = −0.00238; *p* = .03), oils and solid fats (β = −0.00146; *p* = .03) had a negative relationship with log-T/S in all studied occupational classes. A positive relationship was reported between the healthy (β = 0.017; *p* = .2) and traditional dietary pattern (β = 0.012; *p* = .4) with log-T/S, but western pattern identified negative relationship (β = −0.004; *p* = .7). Adherence to a healthy (with consumption whole grains, refined grains, dairy, and cereals) and then traditional pattern with increased consumption of fruits, vegetables and whole grains, fish and dairy products are necessary to prevent TL destruction in all studied occupational classes.

Telomeres are complex nuclei-proteins located at the ends of eukaryotic chromosome, formed by TTAGGG repetitive sequence (Hoxha et al., 2009). Telomere length (TL) is considered as a reliable biomarker of biological aging and age-related chronic diseases (Harte et al., 2012). The loop structures of telomeres are to maintain cellular stability and genomic integrity by preventing irregular recombination of chromosome, DNA degradation by exonuclease, end-to-end fusion, and loss of specific genes due to DNA replication repeat (Karimi, Yunesian, Nabizadeh, Mehdipour, & Aghaie, 2016). Leukocyte telomere length (TL) in the human body is dynamic and typically ranges between 10 and 15 kb (Blackburn, Greider, & Szostak, 2006) and in each cell division cycle about 50 to 200 bp of the TL is progressively shortened, due to the lack of telomeres elongation mechanisms and ends of chromosome replication problem (Karimi et al., 2016). Short TL in leukocyte indicates the risk of cardiovascular and respiratory diseases (Brouilette et al., 2007). Oxidative stress, inflammation, and aging are reported to be associated with TL shortening (Epel et al., 2004). In this condition, cell division capacity is highly increased and TL becomes too short in several stages of cell proliferation. Cells with shorter TL lost the ability of the division and aging, thereby leading to apoptosis and preventing replication of cells (Hohensinner, Goronzy, & Weyand, 2014; Joshu et al., 2015).

The lifestyle factors such as obesity, BMI, smoking, and higher blood and cellular lipids may be associated with both telomere biology and oxidative stress (Buxton et al., 2011; García-Calzón et al., 2014). The associations between foods, dietary patterns and leukocyte TL in previous epidemiological studies have been conflicted (Cassidy et al., 2010; Crous-Bou et al., 2014; Nettleton, Diez-Roux, Jenny, Fitzpatrick, & Jacobs, 2008; Tiainen et al., 2012). Observation and intervention studies identified that adherence to a healthy and Mediterranean dietary patterns has numerous advantages such as reduced chronic diseases and increased chances of health in old age and is associated with longer TL (Strandberg et al., 2011), but other studies did not report this relationship between TL and food groups (Crous-Bou et al., 2014; Gu et al., 2015). Some food components have antioxidant and anti-inflammatory properties, thus, preventing some cancer effects (Shen et al., 2009). Intake of foods containing antioxidants was associated with longer TL (García-Calzón, Moleres, et al., 2015).

Concerning conflicting reports on the effect of the food groups on TL in different communities (Cassidy et al., 2010; Crous-Bou et al., 2014; Nettleton et al., 2008; Tiainen et al., 2012), a part of this conflict can be related to occupational type (Shiels et al., 2011). The type of occupation is one of the major risk factors associated with age-related diseases (Shiels et al., 2011). Regarding the effect of the job on oxidative stress and TL, this study investigates the effect of food intake, dietary patterns, and serum lipids on TL in three occupational classes in a population-based study.

Waste recycling with excessive exposure to a variety of pollutants and higher levels of oxidative markers in the blood resulted in the comparison of the effect of dietary patterns in this job with office-workers that are less exposed to pollutants (Ni, Huang, Wang, Zhang, & Wu, 2014). Therefore the aim of this study was to investigate the effect of food intake and dietary pattern and other confounding factors which affect TL in three classes of occupations (waste-recyclers, office-workers, and other workers) in the urban areas of Tehran.

## Method

### Questionnaire Study

In this study, 300 healthy men of 25 to 40 years old were selected from three occupational classes according to International Standard Classification of Occupations (ISCO) with minor modifications as follows (Porta et al., 2008): (a) managers, professionals, employees, students, elementary occupations such as housework, self-employment, and so on (as office-workers); (b) solid waste-recyclers; and (c) other occupational groups such as service workers, industrial workers, agricultural and fishery workers.

The questionnaire is made up of two parts. The first part is about the demographic information with focus on the residence, workplace, and other confounding factors that might affect TL, such as smoking. The second part is related to the food intake and dietary pattern by food frequency questionnaire (FFQ). The participants’ demographic, age, length of stay in the city, current address, education, previous illnesses, occupation and occupational exposure, and pesticide mixture exposure were asked, and BMI through the measurement of weight and height (kg/m^2^) was calculated and categorized in three groups: 25 (normal), 25 to 30 (overweight), and ≥30 kg/m^2^ (obese) (World Health Organization, 2013). Smoking status was classified into four groups: never-smokers, second-hand smokers, former smokers, and current smokers. The cumulative dose of smoking was computed by the following equation: pack-years = smoked years × packs per day.

### Dietary Analysis

The semiquantitative FFQ designed in the Tehran Lipid and Glucose Cohort Study was employed for assessing the dietary patterns and usual dietary intakes during the past 12 months (Hosseini Esfahani, Asghari, Mirmiran, & Azizi, 2010). The FFQ comprised 148 food items in addition to a standard size of each nutrient and its validity and reliability have been confirmed in 13 districts of Tehran (Hosseini Esfahani et al., 2010). The consumption frequency of each food was questioned from individuals in the last year. Frequency of consumption of any food in terms of frequency of usage per day, week, or month was questioned. The values stated for each food were converted into actual food intake (g/day) using the guide scales.

### Blood Samples and DNA Extraction

After completing the questionnaires, 10 cc blood samples were taken. Quickly, the serum content of about half of the blood samples was separated by centrifuge (5 min at 4000 rpm), kept in glass vials, and maintained in −20°C before testing. Whole blood samples were collected in ethylenediaminetetraacetic acid (EDTA) tubes and stored at −20°C before use. Genomic DNA was extracted from 2 ml of each blood sample by salting-out method (MWer, Dykes, & Polesky, 1988). The concentration of extracting DNA and its quantity and purity was assayed using Nanodrop (Thermo Scientific, Wilmington, DE, USA) and by considering the A260/A280 ratio. All extracted DNA was stored at −70°C until use.

### Lipid Analysis

Serum samples were assessed on the first day of admission of serum lipid levels. Total cholesterol (TC) and triglycerides (TG) were measured by the kits purchased from the PARS-Azmoon Company. Per the study of Bernert et al. 2007 and the existence of a linear relationship between the phospholipids and TC, phospholipids (PL) concentrations were obtained from PL = (0.766 × TC) + 62.3 mg/dl (Bernert, Turner, Patterson, & Needham, 2007). Finally, the total serum lipids (TSL) were estimated by the following equation (Bernert et al., 2007): TSL = (1.494 × TC ) + TG + PL mg/dl.

### Relative Telomere Length

The relative TL was determined with a real-time PCR method-based telomere assay previously described by Cawthon (2002). In summary, real-time PCR was done using SYBR Premix Ex TaqTM kit (Takara) and the primer concentrations for the telomere were 270 nM Tel1 [5’-CGG TTT(GTTTGG)5GTT-3’] and 900 nM Tel2 [5’-GGC TTG(CCTTAC)5CCT-3’] and for single-copy gene primers, 300 nM for 36B4u [5’-CCCATTCTATCATCA ACGGGTACAA-3’] and 500 nM for 36B4d [5’-CAG CAAGTGGGAAGGTGTAATCC-3’] were used. The reaction was performed three times in duplicate wells for each sample using 25 ng/ml of DNA. In each run, three no-template controls alongside other samples and under the same conditions were included for primer-dimer study and correct gene amplification process. Melting curve analysis was used for evaluating the property and verifying the specificity of each run. The standard curve to evaluate the PCR efficiency in each run was utilized by serially diluting one reference DNA sample with deionized water to make six concentrations of DNA ranging from 1.56 to 50 ng/ml. The TL reference value was determined by mixing DNA of 10 randomly selected DNA samples. The TL for each sample was estimated by determining the ratio of the number of telomere repeat copy number (T) to the relative number of 36B4 copies (S) with respect to the same reference DNA sample. The results were expressed in terms of T/S ratios.

### Statistical Analyses

The χ^2^ test was applied to compare the frequency of qualitative independent variables (smoking, education, and years of occupation) in all occupational classes. Furthermore, Analysis of variance (ANOVA) was used for quantitative variables’ [height, weight, BMI, age, residence in Tehran, place of residence (years), mean number of pack-years, occupational class and concentration of TC, TG, PL, and total serum lipids (TSL)] to examine the mean difference between the tertiles of T/S in three occupational groups.

The dietary pattern was determined by factor analysis and then dietary patterns and other effecting factors will be selected by principal components analysis (PCA) with varimax rotation. Afterwards, resulting factors were assessed based on Eigenvalues ≥1 and Scree-plot graph. By observing the factor loading of food items and based on previous studies, four factors extracted were named as healthy, western, traditional, and vegetarian dietary patterns, respectively. The factor loadings of each food patterns were reported in Supplementary Table 1.

Multiple linear regression (MLR) model considering T/S as the response variable was used to investigate the relationship between dependent variables and T/S. Per Shapiro–Wilk test, T/S was not normally distributed (*p* = .00), as a result log-T/S was applied. Two unadjusted and adjusted models were examined based on age, BMI, and smoking status (data of unadjusted model not reported). MLR coefficients were reported for the three job categories (office-workers, waste-recyclers, and other occupational class) based on serum lipids, food group intake, dietary patterns, and other confounding factors. The mean ± SD, 95% confidence interval (95% CI), and median of predicted T/S from MLR and generalized additive model (GAM) were reported. All statistical tests were two-sided, and *p*-values   less than .05 were statistically significant. All statistical analyses were carried out using statistical software R 3.2.2 and SPSS 22.

## Results

**Figure 1.**
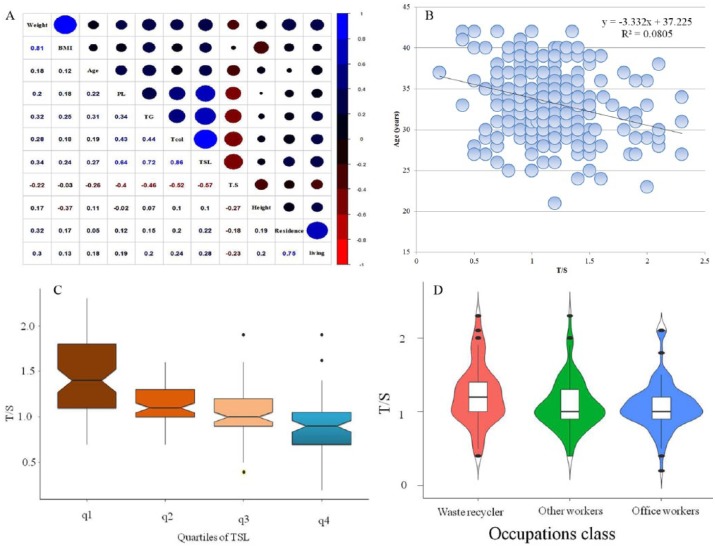
(a) Spearman correlation test, (b) telomere length with age, (c) quartiles of TSL, and (d) occupational class. TSL = total serum lipids.

The average T/S ratio in the study population was 1.13 ± 0.36. Figure 1 reports the average T/S ratio of three occupational classes (*p* = .007). Longer T/S reports more desirable performance status of the body’s cells. The average age of office-workers, waste-recyclers, and other workers were 32.6 ± 4.5, 33.35 ± 3.7, and 34.3 ± 4.3 years, respectively. Increase in age was associated with shortening of T/S during blood sampling (*p* = .02) (Table 1). Approximately 17–24% of the total studied population were smokers in three occupational classes. But no statistical difference was reported between the three groups in terms of smoking (Table 2).

**Table 1.** Characteristics of the Study Participants by Tertiles of T/S.

	Short, mean ± SD			Middle, mean ± SD			Longest, mean ± SD			*p* ^a^
Work	Office-workers	Waste recycling	Other workers	Office-workers	Waste recycling	Other workers	Office-workers	Waste recycling	Other workers	
T/S	0.75 ± 0.2	0.77 ± 0.2	0.78 ± 0.15	1.13 ± 0.12	1.11 ± 0.1	1.1 ± 0.11	1.67 ± 0.28	1.65 ± 0.3	1.61 ± 0.3	.007
Height (cm)	180.66 ± 6.24	180.97 ± 7.4	178 ± 8.6	175.08 ± 8	173.6 ± 8.86	174.92 ± 8.5	175.45 ± 9.6	175.5 ± 13.2	170 ± 1	.49
Weight (kg)	83.14 ± 17.5	83.63 ± 12.4	80.45 ± 14.5	78.38 ± 8.6	76.11 ± 9.62	79.32 ± 12.4	80.7 ± 14.6	70.57 ± 13.3	73.16 ± 1	.35
BMI (kg/m^2^)	25.32 ± 4.6	25.73 ± 4.8	25.74 ± 6	25.69 ± 3.36	25.51 ± 4.56	26.11 ± 4.9	26.17 ± 4.16	22.93 ± 3.46	25.53 ± 5	.54
Age (years)	33.45 ± 5.18	35.06 ± 3.6	35.75 ± 4.2	33.29 ± 4.4	32.81 ± 3.46	34.27 ± 3.8	31.33 ± 3.96	31.5 ± 3.7	32.1 ± 4.8	.02
Residence (years)	18.2 ± 9.1	14.69 ± 9.3	14.59 ± 8	17.83 ± 10.5	10.19 ± 5.38	11.88 ± 7.4	17.17 ± 13.2	6.13 ± 5.4	16.3 ± 12.57	.00
Live in place (years)	12.45 ± 9.25	9.72 ± 6.8	10.97 ± 6.6	13.12 ± 1	6.37 ± 3.26	8.43 ± 5.2	11.1 ± 11.62	5.13 ± 4.2	10.05 ± 11	.00
Mean number of pack-years	23 ± 15.47	40.6 ± 14.3	38.75 ± 13	55.83 ± 5.5	38.15 ± 9.81	37.79 ± 18.9	86.18 ± 12	29.14 ± 7.3	57.5 ± 37.46	.07
TC (mg/dl)	205.05 ± 25.78	203.12 ± 26	200.83 ± 25.7	176.98 ± 29.1	185.28 ± 25.47	186.6 ± 23.1	162.7 ± 22.62	158.57 ± 20.8	170.5 ± 26.2	.04
TG (mg/dl)	172.58 ± 40.95	161.12 ± 30	160.21 ± 32.6	145.05 ± 26.7	145.78 ± 31.52	150 ± 23.3	128.2 ± 47.7	107.68 ± 32.5	124.4 ± 45.4	.79
PL (mg/dl)	216 ± 22.5	216.2 ± 20.6	204.9 ± 21.6	200.56 ± 20.3	208.39 ± 18.78	206.1 ± 17	188.06 ± 2	182.15 ± 14.7	194.8 ± 22.6	.06
TSL (mg/dl)	694.91 ± 79.35	680.77 ± 65.8	665.16 ± 72	610.01 ± 52.6	625.86 ± 74.4	628.54 ± 65	559.4 ± 76.7	526.74 ± 54.7	574 ± 82.5	.24

*Note.* BMI = body mass index; TC = total cholesterol; TG = triglycerides; PL = phospholipids, TSL = total serum lipids.

a p-values from ANOVA between office-workers, waste recycling and other workers.

**Table 2.** Characteristics of the Qualitative Variables of Participants.

	Work, *n* (%)			*p* ^a^	Total
	Office-workers	Waste recycling	Others		
Smoking status (%)					
Never smoke	42 (46.7)	43 (43)	35 (40.7)	−	120 (43.5)
Second-hand smokers	15 (16.7)	17 (17)	20 (23.3)		52 (18.8)
Previously smoking	15 (16.7)	16 (16)	16 (18.6)		47 (17)
Smokers	18 (20)	24 (24)	15 (17.4)		57 (20.7)
Total	90 (100)	100 (100)	86 (100)	.83	276 (100)
Education (%)				−	
Under diploma	4 (4.1)	21 (20.8)	13 (13.7)		38 (13)
Diploma	27 (27.8)	44 (43.6)	36 (37.9)		107 (36.5)
Graduate	33 (34)	27 (26.7)	38 (40)		98 (33.4)
Postgraduate	33 (34)	9 (8.9)	8 (8.4)		50 (17.1)
Total	97 (100)	101 (100)	95 (100)	.00	293 (100)
Years of occupation				−	
<2	16 (16.8)	9 (9)	12 (12.8)		37 (12.8)
2–5	36 (37.9)	61 (61)	32 (34)		129 (44.6)
6–10	22 (23.2)	27 (27)	35 (37.2)		84 (29.1)
11–20	21 (22.1)	3 (3)	15 (16)		40 (13.5)
Total	95 (100)	100 (100)	94 (100)	.00	289 (100)

*Note.* ^a^ *p*-values from χ^2^ squared for categorical variables.

The average T/S of smokers in all groups was 1.16 ± 0.39, that in waste-recyclers, office-workers, and other workers were 1.26 ± 0.34, 1.11 ± 0.42, and 1.1± 0.41, respectively. T/S ratio in never-smokers in all groups was reached (1.09 ± 0.34). T/S ratio of never-smokers in waste-recyclers, office-workers, and other workers were 1.1769 ± 0.42, 1.016 ± 0.24, and 1.08± 0.32, respectively. The average concentrations of total serum lipids (TSL) in office-workers, waste-recyclers, and other workers were 612 ± 83, 628 ± 84.5, and 629 ± 77 mg/dl, respectively (Table 1). With increasing TC, telomere length was significantly reduced in all occupational classes (*p* = .04). Spearman correlation analysis revealed an inverse relationship between age (*r* = −.26), weight (*r* = −.22), BMI (*r* = −.03), years of residence (*r* =−.18), TSL concentration (*r* = −.57), and T/S in all occupational class. Shortening of T/S with age, occupational class, and quartile of TSL is reported in Figure 1a. With increase in TC, TG, PL, and TSL, telomere length was significantly reduced in all occupational classes (*p* = .00). Spearman correlation analysis reported an inverse relationship between age (*r* = −.26), weight (*r* =−.22), BMI (*r* = −.03), years of residence(*r* = −.18), TSL concentration (*r* = −.57), and T/S in all occupational classes. Shortening of T/S with age (Figure 1b), occupational class (Figure 1c), and quartile of TSL is presented in Figure 1d.

**Figure 2.**
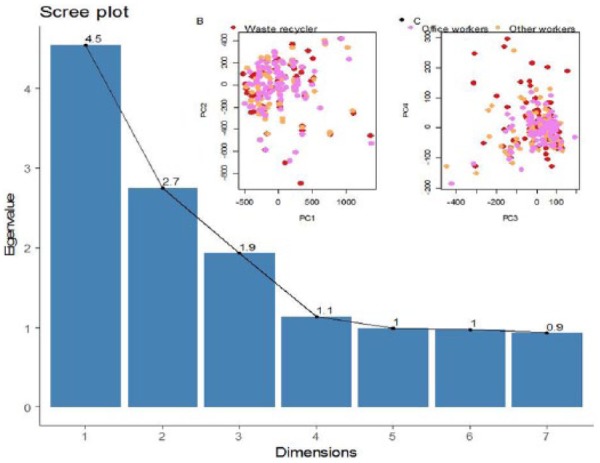
PCA-based analysis of data. (a) Scree-plot (b and c); PCA was applied to separation occupational class.

Per PCA and Scree-plot graph, four dominant dietary patterns of the study were determined (Figure 2a and b). The four patterns stated above explained a total of 64.5% of total variance and the first dietary pattern had the highest contribution. In a healthy diet, whole grains, refined grains, dairy, and cereals had the highest factor-loading, respectively. In the western pattern, animal and solid fat, fish and poultry, salt and spice, processed meats, sweets, dessert, sugar, and refined cereals had the highest factor-loading, respectively. In the traditional pattern, it was fruits and vegetables, whole grains, and dairy products and in the fourth dietary pattern (vegetarian diet) fruits and vegetables had the highest factor-loading, and other foods were not consumed.

Multiple linear regression (MLR) model in food groups identified that whole grains (*p* = .05), refined grains, fruits and vegetables, fish and dairy products were associated with positive correlation with longer T/S; consumption of other food compounds such as meat, produced meats, oils, and fats had a negative relationship with T/S (Table 3). A similar trend was observed in other occupational classes.

**Table 3.** Multiple Linear Regressions Between log-T/S and Intake of Different Food Groups.

	All		Office-workers		Waste-recyclers		Other workers	
	β	*p-value*	β	*p-value*	β	*p-value*	β	*p-value*
(Intercept)	0.4169	.04	−0.7184	.13	0.7089	0	0.6467	.28
Whole grains	0.02312	.05	0.000015	.9	0.0277	.04	0.04701	.08
Refined grains	0.000245	.87	−0.005	.17	0.0021	.22	0.003694	.27
Vegetables and fruits	0.000099	.86	0.00064	.53	0.00062	.38	0.000675	.6
Fish products	0.00016	.24	0.00107	.01	0.0002	.16	0.000196	.52
Dairy products	0.000091	.61	−0.0002	.63	0.000121	.55	−0.000084	.84
Nuts seeds	−0.00072	.06	−0.00036	.69	−0.00085	.04	−0.00042	.68
Meats	−0.00043	.92	−0.00868	.27	−0.008	.12	−0.00067	.94
Produced meats	−0.00238	.03	−0.00238	.3	−0.003	.02	−0.00174	.41
Liquid oils	−0.00034	.55	−0.0016	.3	−0.00015	.8	−0.00173	.29
Solid fats	−0.00146	.03	−0.00036	.83	−0.0015	.04	−0.00345	.08

* Adjusted Model for age (at time of blood sample collection for telomere), education, BMI, smoking status.

The results of coefficients and significant value of MLR model, between T/S and height, weight, BMI, age, residence in Tehran, years of place lived in, years of occupation, pack-years, TC, TG, PL, TSL, and dietary patterns (Healthy, Western, Traditional and Vegetarian) are presented in Table 4. *R*² adjusted value for the overall model is .684.

**Table 4.** Multiple Linear Regressions Between log-T/S and Dietary Pattern and Other Covariate.

	All		Office-workers		Waste-recyclers		Other workers	
	β	*p-value*	β	*p-value*	β	*p-value*	β	*p-value*
(Intercept)	1.717	.25	5.667	.005	3.324	.23	2.637	.59
Height (cm)	−0.00683	.43	−0.03394	.0065	−0.01779	.26	−0.0089	.74
Weight (kg)	0.001655	.86	0.03051	.012	0.01094	.55	0.0056	.84
BMI (kg/m^2^)	−0.00642	.82	−0.08675	.016	−0.04386	.44	−0.0175	.85
Age (years)	−0.00145	.67	0.0108	.01	0.009595	.23	−0.0057	.61
Residence (years)	−0.00027	.89	−0.0022	.29	−0.00101	.87	−0.0052	.49
Live in a place (years)	−0.00143	.62	−0.00622	.06	−0.00782	.23	0.0081	.46
Occupational class	0.037436	.01	0.000017	.99	0.01372	.71	0.02494	.60
Mean number of pack-years	0.000399	.18	0.000709	.00	0.001271	.56	0.00082	.70
TC	−0.00216	.0	−0.00029	.78	0.000691	.61	−0.00387	.07
TG	−0.00146	.002	−0.00175	.01	−0.00309	.006	−0.00138	.41
PL	0.00035	.58	0.000418	.46	0.0000384	.98	0.00008	.97
TSL	−0.0011	.002	−0.00132	.00	−0.00103	.00	−0.00088	.00
Healthy dietary pattern	0.017133	.21	0.002123	.91	−0.00204	.91	0.0281	.59
Western dietary pattern	−0.00424	.79	−0.05059	.13	−0.01646	.57	−0.016	.78
Traditional dietary pattern	0.012512	.45	−0.04081	.17	0.04068	.16	0.01306	.79
Vegetarian diet	0.012989	.33	−0.02224	.14	0.0252	.52	−0.0021	.95

*Note.* BMI = body mass index; TC = total cholesterol; TG = triglycerides; PL = phospholipids; TSL = total serum lipids.

* Adjusted model for age (at time of blood sample collection for telomere), education, BMI, smoking status.

Predicted values of T/S from MLR and GAM are reported in Table 5.

**Table 5.** Predicted T/S Values From Dietary Pattern and Other Covariate by MLR and GAM.

	Predicted values from LRM			Predicted values from GAM		
	Mean ± SD	95% CI	Median	Mean ± SD	95% CI	Median
Total	1.090 ± 0.26	[0.994, 1.204]	1.055	1.092 ± 0.20	[0.917, 1.304]	1.081
Office-workers	1.201 ± 0.31	[0.979, 1.473]	1.146	1.196 ± 0.24	[0.920, 1.560]	1.199
Waste-recyclers	1.069 ± 0.38	[0.747, 1.535]	1.037	1.042 ± 0.19	[0.857, 1.271]	1.068
Other workers	1.046 ± 0.24	[0.858, 8.538]	1.002	1.074 ± 0.26	[0.846, 1.356]	1.048

*Note.* MLR = multiple linear regression; GAM = generalized additive model.

## Discussion

This is the first study that evaluates the relationship between the TL and foods, dietary patterns, and occupational groups in a young male population. Three hundred young male aged 25 to 40 years were compared with each other after grouping in terms of occupational class. Only healthy subjects with no disease and having the least effect on the results of TL were selected. Thus, the effects of aging and chronic diseases on TL were eliminated. Numerous studies have reported that shorter telomeres were associated with age and chronic diseases like dyslipidemia (Aulinas et al., 2015), hypertension (Paik, Kang, Cho, & Shin, 2016), and diabetes (Zhao et al., 2014). Telomere shortening was increased with increase in age, BMI and weight, residence years, and total serum lipids (TSL) (Lynch et al., 2017). TL is dynamic and continuously changing, and its length can be changed in both directions during life (Hovatta et al., 2012). Longer TL was seen after a definite period of observation and intervention studies (Nordfjäll et al., 2009; Svenson et al., 2013). Per the study of Svenson et al. (Svenson et al., 2013), this TL shortening is dependent on the original length of TL. In most studies, TL is reduced by aging (Brümmendorf et al., 2001; Hohensinner et al., 2014; Robertson et al., 2000), but in the study of Strindberg et al., age was not associated with TL (Strandberg et al., 2011). In addition, obesity, BMI, and smoking had a direct relationship with shorter TL (Strandberg et al., 2011). Although the use of a broad range of age in telomere studies is common (Aviv, Valdes, & Spector, 2006), these studies may have been biased and reported TL was not correct. With longer age, obesity, inflammatory reactions, oxidative stress, and other affected risk factors on TL shortening, such as smoking and chronic diseases are usually increased. Per previous reports, higher concentration of blood lipids was associated with TL shortening and atherosclerosis (O’Donnell et al., 2008; Okuda et al., 2002).

In the current study, the type of food intake, diet, and job affected TL. Among all food compounds, greater consumption of grains, fruit and vegetables, fish and dairy products (only in waste recycle) were related to longer TL and, meat, and processed meat were associated with shorter TL (Table 3). Per other studies, whole grains, vegetables, and nuts were inversely associated with age and total mortality-related diseases (Jaiswal McEligot, Largent, Ziogas, Peel, & Anton-Culver, 2006; Knoops et al., 2004; Steffen et al., 2003). This relationship reveals the effect of diet on the aging process of cells (Cherkas et al., 2006). TL shortening is the best index of biological aging of cells. Thus, proper consumption patterns that are influenced by lifestyle can be associated with TL and its related parameters such as inflammation, chronic diseases, and mortality (Lin, Epel, & Blackburn, 2012). Whole grains are considered as a useful component in a healthy diet. The results of this study and that of Cassidy et al. reported that whole and refined grains were associated with longer TL (A. Cassidy et al., 2010). Consumption of whole grains and plant foods due to the present of complex carbohydrates was related to the reduction of blood lipids, obesity, diabetes, cardiovascular disease and enhance the systemic inflammation (Lopez-Garcia et al., 2004; Poppitt et al., 2002). Thus, grains as a useful component in a healthy diet with vegetables, fruits, and smaller amounts of dairy and meat have beneficial health effects on the entire community and improve TL. But in some cross-sectional studies, whole and refined grains in the diet had no positive effect on TL (García-Calzón, Moleres, et al., 2015; Gu et al., 2015).

This study and several others report a positive effect of vegetables and fruits on longer TL, and a direct relationship was observed between TL and the consumption of vegetables, root, pepper, and carrot among the vegetables had the highest relationship with TL (Lee, Jun, Yoon, Shin, & Baik, 2015; Marcon et al., 2012). Per a case-control study having more than 1,600 participants, a healthy lifestyle with fruits and vegetables in diet, in addition to physical activity, low BMI, and lack of smoking were associated with a longer TL (Mirabello et al., 2009). In a cohort study, a direct relationship was observed between vegetables’ consumption and TL length in women which is similar to the results of this study (Tiainen et al., 2012). In a case-control study, this relationship was observed between 300 gastric-cancer patients and 416 controls (Hou et al., 2009). Similar to this study and according to the study of Lee, a direct relationship was identified between the consumption of fruits and TL in middle-aged and adults. In another study, in men, a direct relationship was identified between the consumption of fruits and TL, but this relationship was not identified in women. Another study reported the consumption of the fish, fruits and vegetables are positively associated with TL. Similar to this study and per the study of Lee et al., a direct relationship was identified between the consumption of fruits and TL in middle-aged and adults (Lee et al., 2015). In another study, a direct relationship was reported between the consumption of fruits and TL in men, but this relationship was not identified in women (Tiainen et al., 2012). Another study reported that the consumption of fish, fruits and vegetables are positively associated with TL (Bethancourt, 2014). But some studies such as Gu et al. (Gu et al., 2015) and several other studies reported no significant relationship between vegetables and/or fruits and TL (Gu et al., 2015; Tiainen et al., 2012). Participants in these studies were mainly from certain races (white and Caucasian) and the average age of most of them in these studies such as the study of Chan was very high (Chan, Woo, Suen, Leung, & Tang, 2010). Thus, the results cannot be generalized for all. Vegetables and fruits are the major sources of flavonoids, which have antioxidant property (Freitas-Simoes, Ros, & Sala-Vila, 2016). Nutrients in fruits and vegetables can affect TL through mechanisms that are involved in cellular functions such as DNA repair and maintenance of chromosomes, prevention of methylation of DNA, prevention of inflammation, oxidative stress, and increased telomerase activity (Gu et al., 2015). Anti-inflammatory and antioxidant properties of the materials can reduce the loss of telomere sequences (Gu et al., 2015).

Per Table 3, a negative relationship was identified between the consumption of meat, dairy products (only in office-workers), processed-meat, and TL. Several other studies reported no significant relationship between dairy consumption and TL (Chan et al., 2010; Marcon et al., 2012; Nettleton et al., 2008). Majority of these studies were conducted in different ethnic groups and in both female and male genders. In another study on children, dairy consumption had no effect on TL (García-Calzón, Moleres, et al., 2015). In the study of Song et al., milk consumption was negatively related to TL in healthy women, and fat cheese consumption compared with low-fat cheese was associated with shorter TL (Song et al., 2013). In contrast to these results and similar to this study, Lee demonstrated that higher consumption of dairy products was associated with an increase in TL (Lee et al., 2015). Gu et al., 2015 revealed that dairy consumption was associated with an increase in TL in the non-Hispanic white population (Gu et al., 2015). Meat products are probably one of the highest sources of saturated fatty acids (SFA) (Givens, 2009). Lee et al. reported that the consumption of less red meat and processed meat and higher intake of grains in Korean middle-aged and adults were associated with longer TL (Lee et al., 2015). Another study on 840 adults indicated that the consumption of processed meat was inversely associated with TL (Nettleton et al., 2008). In the study of Betancourt et al., processed meat, grilled meat, oil, and beverages were associated with shorter TL (Bethancourt, 2014). Per other studies, high intake of processed meat leads to the formation of inflammatory mediator compounds and increases the risk of numerous cancers such as breast cancer (Cho et al., 2006), diabetes (Pan et al., 2011) and cardiovascular diseases (CVD) (Micha, Wallace, & Mozaffarian, 2010). All of these such as TL are age-related diseases. In an experimental study on mice, it was identified that there was reduction in TL when mice were fed with beef for 4 weeks, and dose–response relationship was also found (O’Callaghan et al., 2012). The principal mechanism of the biological relationship between foods and TL is vague, and inflammatory reactions and oxidative pathways may be involved (García-Calzón, Zalba, et al., 2015; Szebeni et al., 2014). Nevertheless, this relationship was not identified in several other studies (Crous-Bou et al., 2014; Gu et al., 2015; Marcon et al., 2012). Although, in most of these studies, the sample size was large and there were different ethnicities, the studied populations were mostly women and/or elderly men.

In this study, an inverse relationship was identified between fat intake and TL. Tiainen et al. reported that men significantly had a shorter TL when total fat, SFA, and butter were consumed (Tiainen et al., 2012). In Song et al.’s study, butter consumption was inversely associated with TL after adjusting the model based on the age (Song et al., 2013). Nettleton et al. in his study, identified that a dietary pattern with fat and processed meat was inversely associated with TL (Nettleton et al., 2008). But in the study of Macron et al., this relationship was not found (Marcon et al., 2012). In the study of Farzaneh et al. it was identified that comprehensive changes in men’s lifestyle, including reduction of fatty foods, intake of herbal food, consumption of omega-3 supplements, soy, vitamins C and E over a period of 3 months increased telomerase activity and finally resulted in longer TL (Farzaneh-Far et al., 2010). In the study of Cassidy et al., an inverse relationship was identified between fat intake of polyunsaturated fatty acids (PUFA) and TL in women (Cassidy et al., 2010). In another study, reduced low-density lipoprotein plasma concentration was associated with increased telomerase activity and reduced psychological distress (Ornish et al., 2008). Thus, the intake of total fat and saturated fat is associated with shorter TL. Long-chain PUFA diet, which has antioxidant and anti-inflammatory properties, is useful to prevent the destruction of TL (Freitas-Simoes et al., 2016).

Although TL is under the control of genetic characteristics and individual differences (Graakjaer et al., 2004), it is also affected by environmental factors and job (Fujishiro, Diez-Roux, Landsbergis, Jenny, & Seeman, 2013). Work-related stress occurs when the demands of the work environment exceed the worker’s capacity (Ahola et al., 2012). The side effect of this type of oxidative stress is cell aging (Epel et al., 2004). TL shortening is a part of the response to chronic stress or psychological stress by the body’s cells (Ahola et al., 2012). In both unadjusted and adjusted models based on the age, BMI, and smoking, waste-recycling significantly had shorter TL than office-workers. Per other studies, the degree of inflammation and oxidative status is different among different ethnic and occupational groups (Needham et al., 2013). TL shortening in waste recycling can be caused by high job stress (Fujishiro et al., 2013). Population-based studies indicated that waste recycling had higher levels of oxidative stress markers in the blood compared to other people in the community, due to excessive exposure to pollutants (Chen, Dietrich, Huo, & Ho, 2011). DNA oxidative damage indices such as 8-hydroxy-2-deoxyguanosine and the presence of leukocytes in urine and systemic oxidative stress indices, including blood malondialdehyde, lipid-peroxide, and total-urinary biopyrrins in waste-recyclers’ blood was very high (Yoshida et al., 2003). Low concentrations of tocopherol antioxidants (Ekor & Odewabi, 2014) and higher levels of inflammatory indices (Kuijer, Sluiter, & Frings-Dresen, 2010) such as IL-6 were identified in their blood (Kuijer et al., 2010). Diet and lifestyle can cause inflammation, oxidative stress, and increased psychological pressure, and all these factors affect TL (Crous-Bou et al., 2014). Other nonbiological mechanisms and confounding factors or exposure to environmental pollutants’ may affect TL shortening in waste-recyclers.

Evidence suggests that the food health depends on the total diet composition, rather than its components, and synergistic-interactions may exist between different foods. Per PCA of food data, four healthy, western, traditional, and vegetarian dietary patterns were identified. Per Table 4, a positive relationship was identified between the healthy dietary pattern and TL, and a negative relationship was identified between western pattern and TL in all occupational groups. The results revealed that the traditional pattern and herbal pattern, respectively were associated with increased and reduced TL. This negative relationship between TL and western pattern can be attributed to the consumption of low healthy foods and high levels of undesirable foods (such as produced meats and high fat content). Intake of saturated fatty acids in the western pattern is associated with insulin resistance and metabolic diseases (Isharwal, Misra, Wasir, & Nigam, 2009). In previous studies, a healthy or Mediterranean dietary pattern has been associated with reduced obesity and longer TL (Boccardi et al., 2013; Crous-Bou et al., 2014; Gu et al., 2015). The beneficial effects of healthy pattern are due to the variety of vitamins, minerals, phytochemicals, and fiber. High consumption of vegetables reduces visceral fat and risk factors for type 2 diabetes (Mozaffarian et al., 2010). Dairy intake is associated with reduced insulin resistance and dyslipidemia (Mozaffarian et al., 2010). The results of a cohort study revealed a strong relationship between a healthy dietary pattern and reduced markers of inflammation in adults (Greenlee, Arbuckle, & Chyou, 2003). In Nettleton et al.’s study, western pattern with higher consumption of fats and processed meat was associated with shorter TL (Nettleton et al., 2008). In Lopez et al.’s study, western pattern with high consumption of processed meats, sweets, chips, and refined grains was associated with increased inflammatory markers (Lopez-Garcia et al., 2004). During 10 years follow-up of the samples by Lee et al. using FFQ, it was observed that prudent pattern with high intake of whole grains, seafood, legumes, vegetables, and seaweed was associated with longer TL and western pattern with high consumption of refined grains, processed meat, and beverages was associated with a shorter TL (Lee et al., 2015). Another study with 520 participants after 5 years of monitoring reported a negative relationship between Western pattern, by shortening TL and inflammatory reactions in the elderly and those prone to cardiovascular diseases (García-Calzón, Moleres, et al., 2015). Mediterranean pattern with less intake of saturated fat, meat, and the consumption of vegetarian foods was related to longer TL in healthy women (Crous-Bou et al., 2014). Furthermore, per Boccardi et al. study, adherence to the Mediterranean (Healthy) pattern was associated with a longer TL when compared to the other patterns (Boccardi et al., 2013).

With the aim of fixing the problems of previous studies, in this study, the male’s age of individuals was selected in the range of 25 to 40 years and several confounding factors affecting TL were eliminated. As a result, the statistical power for detecting the relationships between dietary factors and telomere length was increased. Another strong point is the examination of the effect of dietary patterns on three different occupational groups. All samples were population-based and TL was measured using real-time PCR in which a small amount of DNA was required compared to the Southern blot method and it can be used in most of the epidemiological studies (Boccardi et al., 2013; Crous-Bou et al., 2014; Gu et al., 2015).

## Conclusion

In conclusion, change and intervention in a diet is a powerful tool for preventing and delaying chronic diseases. With a thorough understanding of the mechanisms affecting the cellular age, inflammation, oxidative stress, apoptosis, and their relationship with TL, the prevention of numerous diseases can be addressed. The results of this study demonstrated that an inverse relationship was identified between TL and weight, BMI, age, and TSL. High serum lipid concentration may be associated with systemic inflammation and atherosclerosis and may lead to oxidative stress, resulting in telomere shortening. Adherence to a healthy diet with increased consumption of fruits, vegetables, whole grains, and white meat (fish) is necessary to prevent TL destruction and increase life span. According to this study, adherence to a healthy and then traditional diet was significantly associated with an increase in TL in all occupational groups. But the western dietary pattern was associated with shorter TL. Further well-design cohort studies with larger sample size are necessary to survey the effect of the consumption of red and processed meat and dairy products on TL.

This study was cross-sectional and no time relationship has been examined between food groups and TL. Hence, TL reduction due to food intake over the time is unknown. Although the validity and reliability of this method have been investigated in several studies (Hosseini Esfahani et al., 2010), data were collected by using FFQ, and food intake is dependent on the mind of an individual. Therefore, the possibility of error existed. In the next cohort studies, the use of biological markers of nutrient intake to assess the food intake is recommended. Also, investigating the relationship between dietary factors with telomerase activity and markers of inflammation will provide further information.
